# Simultaneous inhibition of Vps34 kinase would enhance PI3Kδ inhibitor cytotoxicity in the B-cell malignancies

**DOI:** 10.18632/oncotarget.10650

**Published:** 2016-07-18

**Authors:** Xiaochuan Liu, Aoli Wang, Xiaofei Liang, Juanjuan Liu, Fengming Zou, Cheng Chen, Zheng Zhao, Yuanxin Deng, Hong Wu, Ziping Qi, Beilei Wang, Li Wang, Feiyang Liu, Yunhe Xu, Wenchao Wang, Stacey M. Fernandes, Richard M. Stone, Ilene A. Galinsky, Jennifer R. Brown, Teckpeng Loh, James. D. Griffin, Shanchun Zhang, Ellen L. Weisberg, Xin Zhang, Jing Liu, Qingsong Liu

**Affiliations:** ^1^ Department of Chemistry, University of Science and Technology of China, Anhui, Hefei, 230036, P. R. China; ^2^ High Magnetic Field Laboratory, Chinese Academy of Sciences, Hefei, 230031, Anhui, P. R. China; ^3^ University of Science and Technology of China, Anhui, Hefei, 230036, P. R. China; ^4^ CHMFL-HCMTC Target Therapy Joint Laboratory, Hefei, 230031, Anhui, P. R. China; ^5^ Department of Medical Oncology, Dana-Farber Cancer Institute, Harvard Medical School, Boston, MA 02115, USA; ^6^ Hefei Cosource Medicine Technology Co. LTD. Hefei, 230031, Anhui, P.R.China; ^7^ Hefei Science Center, Chinese Academy of Sciences, Hefei, 230031, Anhui, P. R. China

**Keywords:** PI3Kδ, Vps34, combination, chronic lymphatic leukemia, acute myeloid leukemia

## Abstract

PI3Kδ has been found to be over-expressed in B-Cell-related malignancies. Despite the clinical success of the first selective PI3Kδ inhibitor, CAL-101, inhibition of PI3Kδ itself did not show too much cytotoxic efficacy against cancer cells. One possible reason is that PI3Kδ inhibition induced autophagy that protects the cells from death. Since class III PI3K isoform PIK3C3/Vps34 participates in autophagy initiation and progression, we predicted that a PI3Kδ and Vps34 dual inhibitor might improve the anti-proliferative activity observed for PI3Kδ-targeted inhibitors. We discovered a highly potent ATP-competitive PI3Kδ/Vps34 dual inhibitor, PI3KD/V-IN-01, which displayed 10-1500 fold selectivity over other PI3K isoforms and did not inhibit any other kinases in the kinome. In cells, PI3KD/V-IN-01 showed 30-300 fold selectivity between PI3Kδ and other class I PI3K isoforms. PI3KD/V-IN-01 exhibited better anti-proliferative activity against AML, CLL and Burkitt lymphoma cell lines than known selective PI3Kδ and Vps34 inhibitors. Interestingly, we observed FLT3-ITD AML cells are more sensitive to PI3KD/V-IN-01 than the FLT3 wt expressing cells. In AML cell inoculated xenograft mouse model, PI3KD/V-IN-01 exhibited dose-dependent anti-tumor growth efficacies. These results suggest that dual inhibition of PI3Kδ and Vps34 might be a useful approach to improve the PI3Kδ inhibitor's anti-tumor efficacy.

## INTRODUCTION

PI3Kδ belongs to the Class I PI3K family, which also consists of PI3Kα, β and γ, and is predominantly expressed in leukocytes.[1] Deregulation of PI3Kδ, for example over-expression, has been found in B-cell malignancies such as Chronic Lymphatic Leukemia (CLL), indolent non-Hodgkin Lymphoma and Acute Myeloid Leukemia (AML).[2, 3] Constitutive activation of B-cell receptor (BCR) or proliferation and survival factors present in bone marrow and lymph node microenvironment may activate the PI3Kδ-mediated signaling pathway, which will lead to aberrant cell proliferation, survival and differentiation.[4] The seminal discovery of the PI3Kδ selective inhibitor, CAL-101 (Idelalisib), and its successful clinical application toward CLL and Indolent NHL, have validated PI3Kδ as a drug discovery target.[5] However, both preclinical and clinical tests have shown that inhibition of PI3Kδ alone only exerted limited cytotoxicity against the transformed cells directly, but rather inhibited cell survival by interfering with the microenvironment, such as by blocking the cytokines TNF-α, IL-6, etc to prevent the leukemic cells from circulating back to the lymph nodes and bone marrow for the further proliferation.[6] In order to improve clinical efficacy, various combination therapies have been suggested such as those involving in chemotherapy, and other signaling pathway inhibitors including MEK, BRAF, MYC, PARP, BCL-2 and autophagy.[7] Among them, the autophagy signaling pathway has attracted special attention since inhibition of the PI3K/AKT/mTOR signaling pathway is known to induce autophagy, which could provide a pro-survival mechanism for the cancer cells to escape apoptosis.[8] In addition, there is evidence that treatment of CLL cells with CAL-101 induces autophagy.[9] Therefore, simultaneous inhibition of PI3Kδ and autophagy might enhance the clinical efficacy of PI3Kδ inhibitors.

Vps34 belongs to Class III PI3K family and has been shown to be essential for autophagy initiation and progression. It is thought that VPS34 kinase can synthesize and deposit the phophatidylinositol-3-phosphate (PtdIns(3)P) on the autophagosome formation site, and hence inhibition of the Vps34 would block the autophagosome formation process and result in prevention of autophagy.[10] Recently, the discovery of several selective Vps34 inhibitors, such as Vps34-IN-1, PIK-III, SAR405, has clearly proven this.[11–14] In addition, the combination of the Vps34 specific inhibitor, SAR405, with the mTOR inhibitor, everolimus, which can induce the autophagy, has been shown to be synergistic in renal cell carcinoma cell lines.[12]

Given the possible protective effects of autophagy induced by PI3Kδ inhibition and the structural similarity between PI3Kδ (Class I PI3K) and Vps34 (Class III PI3K), we postulated that development of a selective PI3Kδ and Vps34 dual inhibitor might enhance the cytotoxic effect of PI3Kδ inhibition on B-cell malignances, such as CLL and AML. This led to the discovery of a highly selective PI3Kδ/Vps34 dual inhibitor, PI3KD/V-IN-01, which displays superior anti-proliferative activity against B-Cell malignant cells *in vitro* and *in vivo*.

## RESULTS

### PI3KD/V-IN-01 is a highly selective and potent PI3Kδ/Vps34 dual inhibitor

**Figure 1:**
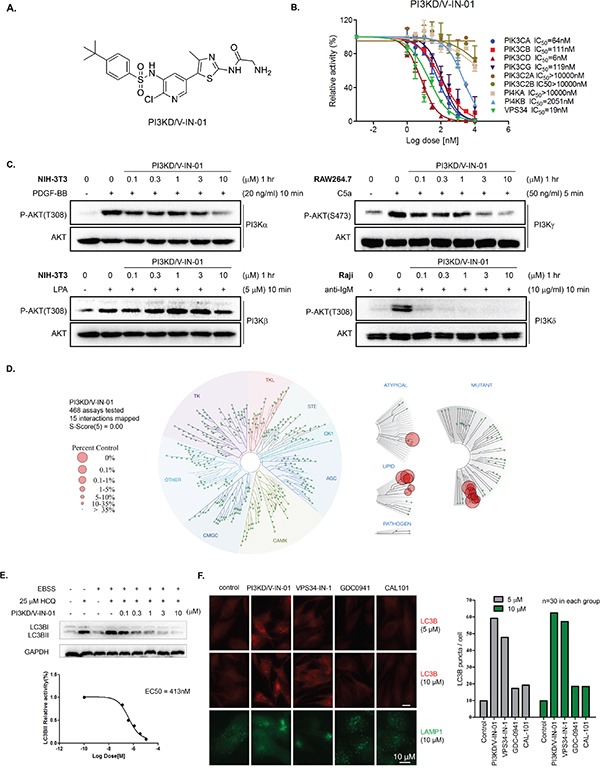
Biochemical and pharmacological characterization of PI3KD/V-IN-01 **A.** Chemical structure of PI3KD/V-IN-01. **B.** ADP-Glo™ Biochemical IC50 determination of PI3KD/V-IN-01 against a panel of PI3K-related kinases. **C.** Determination of the inhibitory effect of PI3KD/V-IN-01 against class I PI3Ks in the cellular context. Specifically, PI3Kα in NIH-3T3 cells with PDGF-BB stimulation; PI3Kβ in NIH-3T3 cells with LPA stimulation; PI3Kγ in RAW264.7 cells with C5a stimulation; PI3Kδ in Raji cells with anti-IgM stimulation. **D.** Selectivity profile of PI3KD/V-IN-01 in the DiscoveRx's KinomeScan™ platform. **E.** Effect of PI3KD/V-IN-01 on autophagy in HeLa cells using co-culture of EBSS and HCQ (25 μM) and investigating LC3BII expression. **F.** Immuno-fluorescent imaging analysis of the effect of PI3K inhibitors on LC3BII expression in HeLa cells and of LAMP1 expression in HeLa cells treated with PI3K inhibitors.

A focused medicinal chemistry approach based on an aminothiazole scaffold led to a highly potent PI3Kδ/Vps34 dual inhibitor, PI3KD/V-IN-01. (chemical structure shown in Figure 1A) In the ADP-Glo™ biochemical assay with purified PI3K isoform proteins, PI3KD/V-IN-01 displayed an IC_50_ of 6 nM against PI3Kδ and 19 nM against VPS34. (Figure 1B) Among other PI3Ks, it exhibited an IC_50_ of 64 nM against Class I PI3K PI3Kα, 111 nM against PI3Kβ, 119 nM against PI3Kγ. In contrast, the drug showed little activity against Class II PI3Ks and PI4Kα/β. A kinetic study with varied ATP concentrations showed that PI3KD/V-IN-01 was an ATP competitive inhibitor against both PI3Kδ and Vps34. (Supplemental Figure 1A, 1B) Further characterization of PI3KD/V-IN-01 in the cellular context for the class I PI3Ks demonstrated that it potently inhibited anti-IgM- stimulated PI3Kδ activity in Raji cells with an EC_50_ of 11 nM, however exhibited far less activity against PI3Kα (EC_50_: 2966 nM), PI3Kβ (EC_50_: >3000 nM) and PI3Kγ (EC_50_: 186 nM). (Figure 1C and Table 1) Further characterization of the selectivity profile in the DiscoveRx's KinomeScan™ platform showed that PI3KD/V-IN-01 (1 μM) is highly selective and did not potently bind to any of other protein kinases (S score (5)=0.00). (Figure 1D) In addition to Class I PI3K family kinases, PI3KD/V-IN-01 also strongly bound to the structurally similar mTOR kinase (% control number =0). However, the activity-based *in vitro* IP kinase assay demonstrated only modest inhibition of mTORC1 kinase by PI3KD/V-IN-01 (EC_50_ of 1700 nM), which is much less potent than activity displayed against PI3Kδ and vps34 kinases. (Supplemental Figure 3) In HeLa cells, PI3KD/V-IN-01 effectively prevented LC3BII accumulation in the presence of EBSS (Earle's balanced salt solution) and HCQ (hydrochloroquine) with an EC_50_ of 413 nM. (Figure 1E) In addition, an immunofluorescence experiment showed that PI3KD/V-IN-01 increased LC3B puncta in HeLa cells in a dose-dependent way, which was similar to the Vps34 specific inhibitor Vps34-IN-1, but not for the PI3Kδ inhibitor CAL-101 or pan-PI3K inhibitor GDC-0941. (Figure 1F and Supplemental Figure 2) This is not surprising as HeLa cells express Vps34 but not PI3Kδ. In accordance with the established function of Vps34 in membrane trafficking [16, 17], the lysosome marker LAMP1 localization was affected by PI3KD/V-IN-01 and Vps34-IN-1, however not GDC-0941 or CAL-101. (Figure 1F) These biochemical and cellular data demonstrate that PI3KD/V-IN-01 was a highly potent and selective PI3Kδ/Vps34 dual inhibitor.

**Table 1:** Quantification of PI3KD/V-IN-01 EC50 against class I PI3Ks

Cellular EC50 (nM)	PI3Kα	PI3Kβ	PI3Kγ	PI3Kδ
PI3KD/V-IN-01	2966	>3000	186	11

### PI3KD/V-IN-01 exhibits anti-proliferative activity against B-cell-related cancer cell lines

We next tested PI3KD/V-IN-01 in a panel of B-cell-related cancer cell lines, including AML, CLL, Burkitt lymphoma and B-cell lymphoma. (Table 2) PI3KD/V-IN-01 was more potent than the selective PI3Kδ inhibitor, CAL-101, and selective Vps34 inhibitor, Vps34-IN-1, against most of the cell lines, however less potent than the pan-PI3K inhibitor, GDC-0941. Specifically, CAL-101 did not effectively inhibit the majority of the cell lines tested, except OCI-AML-3 (AML, GI50: 2.4 μM). Interestingly, Vps34-IN-1 itself showed micro molar potencies against most of cell lines, including two CLL cell lines (HS505T and MEC-1), the latter of which poorly responded to CAL-101, GDC-0941 and PI3KD/V-IN-01 (GI50: >10 μM). What is worthy to note is that PI3KD/V-IN-01 was most potent against several FLT3-ITD-positive cell lines (GI50 below 1μM), including MV4-11, MOLM-13 and MOLM-14. PI3KD/V-IN-01 inhibited colony formation of OCI-AML-2 (AML, EC_50_ 145 nM), OCI-AML-3(AML, EC_50_: 1001 nM), MV4-11 (AML, EC_50_: 124 nM) and MEC-2 (CLL, EC_50_: 1579 nM). (Supplemental Figure 4)

**Table 2:** PI3K inhibitor anti-proliferative effect against a panel of B-cell related cancer cell lines

GI_50_(μM)	Cell type	CAL-101	GDC-0941	PI3KD/V-IN-01	VPS34-IN-1
Primary target	/	PI3Kδ	pan-PI3Ks	PI3Kδ/Vps34	Vps34
U937	AML(FLT3wt)	>10	1.6	2.2	2.1
CMK	AML(FLT3wt)	>10	0.3	2	5.9
NB4	AML-3 (FLT3wt)	>10	1	1.4	6.4
HL-60	AML (FLT3 wt)	>10	0.16	2.7	0.51
OCI-AML-3	AML(FLT3wt)	2.4	0.73	2.4	3.6
OCI-AML-2	AML(FLT3wt)	>10	2	1	6.6
NOMO-1	AML (FLT3wt)	>10	1	2.3	1.8
SKM-1	AML(FLT3wt)	>10	0.3	1	6.4
MOLM-14	AML (FLT3-ITD)	7.8	0.3	0.51	7.8
MOLM-13	AML(FLT3-ITD)	>10	0.15	0.28	0.7
MV4-11	AML (FLT3-ITD)	>10	1.4	0.78	2.4
HEL	AML(FLT3wt)	>10	>10	>10	1.7
HT	B-cell lymphoma	>10	0.5	1.9	1.4
Ramos	Burrkit lymphoma	>10	2.1	5.4	3.3
Namalwa	Burrkit lymphoma	>10	0.39	1.3	8.1
MEC-2	CLL	>10	>10	2.4	3.1
Hs 505T	CLL	>10	>10	>10	3
MEC-1	CLL	>10	>10	>10	3.7

### PI3KD/V-IN-01 suppresses the PI3Kδ-mediated signaling pathway, interferes with autophagy and arrests cell cycle progression in AML and CLL cell lines

**Figure 2:**
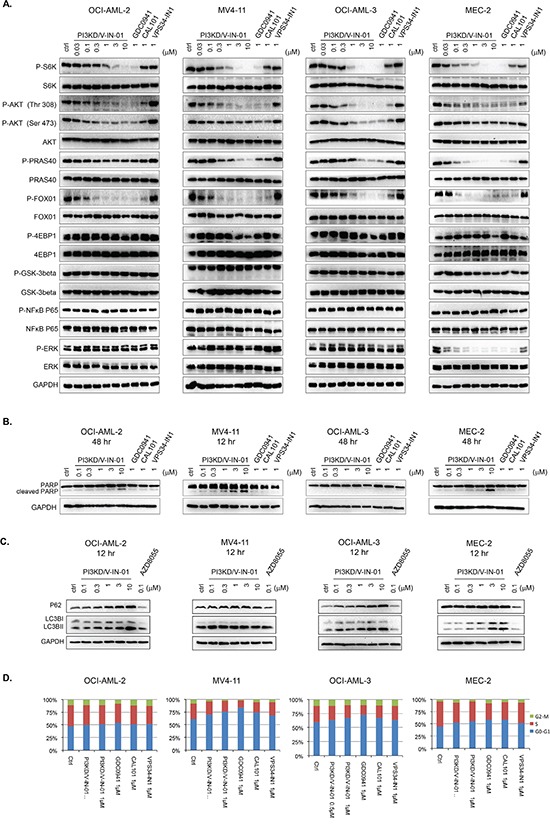
Effect of PI3KD/V-IN-01 on cellular signaling, cell cycle progression, and autophagy **A.** Effect of PI3KD/V-IN-01 on PI3Kδ- mediated signaling pathways in OCI-AML-2 (AML), MV4-11 (AML), OCI-AML-3(AML) and MEC-2 (CLL) cell lines. **B.** Apoptotic effect of PI3KD/V-IN-01 in OCI-AML-2, MV4-11, OCI-AML-3 and MEC-2 cells. **C.** Autophagy interruption effect of PI3KD/V-IN-01 in OCI-AML-2, MV4-11, OCI-AML-3 and MEC-2 cells. **D.** Cell cycle progression effect of PI3KD/V-IN-01 in OCI-AML-2, MV4-11, OCI-AML-3 and MEC-2 cells.

We next examined the effect of PI3KD/V-IN-01 on the PI3Kδ-mediated signaling pathway in OCI-AML-2(AML), OCI-AML-3(AML), MV4-11(AML), and MEC-2 (CLL) cell lines. (Figure 2A) For all four cell lines, PI3KD/V-IN-01 treatment led to inhibition of pAKT(T308, S473) and downstream pFOXO1, pPRAS40 and pS6K. Phosphorylation of 4EBP1 was not inhibited in any of the cell lines, which supports the notion that PI3KD/V-IN-01 does not bear activity against mTOR kinase: this is in accordance with results of the *in vitro* IP kinase assay. Activity of the downstream AKT kinase mediator, GSK3β, and downstream transcription regulator, NFκB, were not affected in any of the cell lines. What is noteworthy is that in MEC-2 cells, pERK was significantly inhibited by PI3KD/V-IN-01, GDC-0941 and CAL-101, but this phenomenon was not observed in OCI-AML-2, OCI-AML-3 and MV4-11 cells. These results demonstrate that PI3Kδ is effectively inhibited in the cellular context, however differential responsiveness of certain signaling molecules to the inhibitors tested suggest that these cells might rely on different genetic/signaling network backgrounds.

We next examined the effect of PI3KD/V-IN-01 on induction of apoptosis. Interestingly, only for MV4-11 was apparent PARP cleavage observed, which clearly suggests drug-induced apoptosis of this line. (Figure 2B) For OCI-AML-2 and MEC-2, there was only a modest induction of apoptosis (evident at 3 μM to 10 μM), and for OCI-AML-3, no apoptosis was observed, even at concentrations up to 10 μM. Of relevance, a concentration-dependent increase in levels of the autophagy markers, LC3BII and p62, was observed in OCI-AML-2, OCI-AML-3 and MEC-2. (Figure 2C) However, this phenomenon was not observed for MV4-11 cell line. Cell cycle analysis showed that after 48 h treatment with PI3KD/V-IN-01, MV4-11, OCI-AML-3 and MEC-2 cells could be arrested in the G0/G1 phase, while there was no apparent cell cycle inhibition observed for drug-treated OCI-AML-2 cells. (Figure 2D)

### Combination of CAL-101 and Vps34-IN-1 recapitulates the dual inhibitory effect of PI3KD/V-IN-01

**Figure 3:**
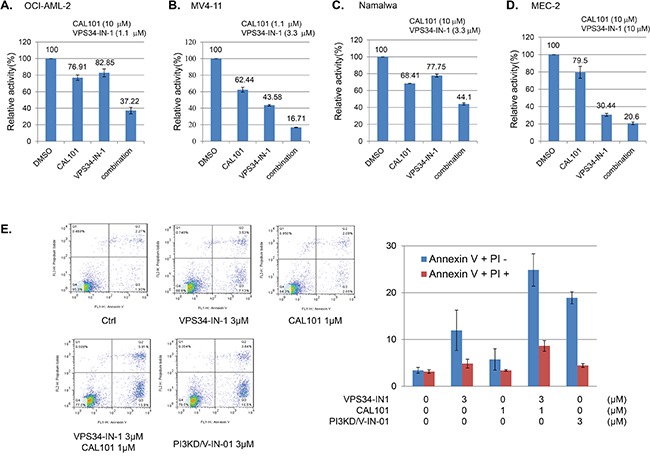
Combinatorial effect of PI3Kδ selective inhibitor CAL-101 and Vps34 selective inhibitor VPS34-IN-1 Combination of CAL-101 and Vps34-IN-1 against OCI-AML-2 **A.**, MV4-11 **B.**, Namalwa **C.**, and MEC-2 **D. E.** FACS PI/Annexin V double staining analysis showing effect of the drug combination on induction of apoptosis.

We next investigated whether or not the combination of the selective PI3Kδ inhibitor, CAL-101, and selective Vps34 inhibitor, VPS34-IN-1, was able to mimic the anti-proliferative effect of dual PI3Kδ/Vps34 inhibitor, PI3KD/V-IN-01. The results suggest that for the OCI-AML-2 cells (AML), 1.1 μM Vps34-IN-1 (treatment with which leads to 82.8% inhibition of cell growth/viability) and 10 μM of CAL-101 (treatment with which leads to 76.9% inhibition of cell growth/viability) would together lead to 37.2% inhibition. (Figure 3A) For MV4-11 cells (AML), 3.3 μM of Vps34-IN-1(treatment with which leads to 43.5% inhibition of cell growth/viability) and 1.1 μM of CAL-101 (treatment with which leads to 62.4% inhibition of cell growth/viability) could together synergistically lead to 16.7% inhibition. (Figure 3B) Similarly, in Namalwa (Burrkit lymphoma) and MEC-2 cells (CLL), 10 μM of Vps34-IN-1 and 3.3 and 10 μM of CAL-101, respectively, together synergistically caused significant inhibition of cell growth/viability. (Figure 3C and 3D) We also investigated the extent of drug-induced apoptosis following drug combination treatment in MV4-11 cells, as PI3KD/V-IN-01 was observed to lead to substantial induction of apoptosis. (Figure 2B) The results suggest that 1 μM of CAL-101 and 3 μM of Vps34-IN-1 significantly increased the percentage of cells undergoing apoptotic cell death, similar to what was observed with 3 μM of PI3KD/V-IN-01. (Figure 3E)

### PI3KD/V-IN-01 inhibited growth of CLL patient primary cells and AML primagraft cells and displayed anti-tumor activity in an MV4-11-inoculated xenograft mouse model

**Figure 4:**
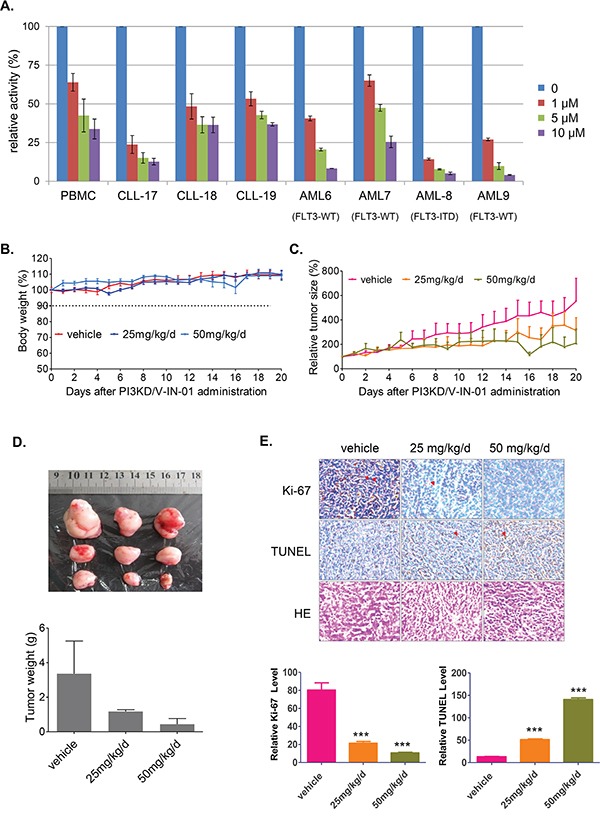
Effect of PI3KD/V-IN-01 on CLL and AML patient primary cells and an MV4-11 cell-inoculated xenograft mouse model **A.** Anti-proliferative effect of PI3KD/V-IN-01 on patient CLL primary cells and AML primagrafts cells. **B.** The effect of PI3KD/V-IN-01 in an MV4-11-inoculated mouse xenograft model: measurement of body weight. **C, D.** The anti-tumor effect of PI3KD/V-IN-01 in an MV4-11- inoculated mouse xenograft model. **E.** Immunohistochemistry staining of Ki67, TUNEL and HE to look at the tumor cell proliferation and apoptosis in the tumor tissues.

PI3KD/V-IN-01 inhibited the growth of primary CLL cells, with approximately 50% of cell proliferation inhibited at 1 μM following 72 hours. (Figure 4A and supplemental Table 1 for the patient information) In addition, PI3KD/V-IN-01 inhibited the growth of AML primagraft cells ex vivo following 72 hours. Of interest, FLT3-ITD positive primary AML cells were observed to be particularly sensitive to PI3KD/V-IN-01, with over 85% inhibition observed at 1μM. (Figure 4A)

Given that FLT3-ITD-positive MV4-11 cells and FLT3-ITD-positive primary AML cells were observed to be especially sensitive to PI3KD/V-IN-01 (see Table 1), we decided to investigate the *in vivo* effects of PI3KD/V-IN-01 using an *in vivo* model of mutant FLT3-positive leukemia. A dose of 50 mg/kg/day administered intraperitoneal almost completely suppressed the *in vivo* growth of inoculated MV4-11 cells with a TGI (Tumor growth inhibition) of 86.8%, with no apparent effects on body weight, suggesting a lack of toxicity. (Figure 4B, 4C and 4D) Immunohistochemistry study showed that the cell proliferation was dose-dependently inhibited (Ki67 stain) and apoptosis was dose-dependently increased (TUNEL stain). (Figure 4E)

## DISCUSSION

The seminal discovery of the first highly selective PI3Kδ inhibitor, CAL-101, has greatly advanced progress in treatment of B-cell related malignancies. However, despite the promising clinical success with CAL-101 as a single agent, cytotoxicity against the cancer cell itself has limited its overall efficacy. Autophagy may be one of the mechanisms whereby cells overcome the activity of PI3Kδ inhibitors such as CAL-101. Here, through the discovery of a highly selective PI3Kδ/Vps34 inhibitor PI3KD/V-IN-01, which can efficiently block the PI3Kδ mediated signaling pathway and autophagy, we found that dual inhibition of PI3Kδ and Vps34 greatly enhances the anti-proliferative effects of single PI3Kδ and Vps34 inhibitors in B-cell cancers such as AML, CLL, and B-NHL. This dual inhibition can be recapitulated by the combination of PI3Kδ and Vps34 selective inhibitors individually. Our results further prove that autophagy is indeed an important factor leading to protection of leukemic cells from death upon PI3Kδ inhibitor treatment, and simultaneous inhibition of both targets potentiates anti-leukemic activity. In addition, given the complexities of drug-drug interactions and increased risk of toxicity resulting from this, discovery of a carefully controlled single agent, multi-targeted drug is preferable.

Interestingly, FLT3-ITD positive cell lines, such as MV4-11, MOLM-13, and MOLM-14, and FLT3-ITD positive primary AML cells are much more sensitive to PI3KD/V-IN-01 inhibition than wt FLT3 AML cell lines and primagrafts. However, this trend was not observed for either of the PI3Kδ selective inhibitor, CAL-101, or the Vps34 selective inhibitor, Vps34-IN-1, or the pan-PI3K inhibitor GDC-0941. These cells are dependent on FLT3-ITD for growth and did not seem to rely on autophagy for escape from cell death upon PI3Kδ inhibitor treatment. Considering the high selectivity of PI3KD/V-IN-01 revealed in KinomeScan™ profiling, it is unlikely that the other kinase targets contribute to its efficacy. One possible reason is that PI3Kδ is a downstream mediator of FLT3-ITD activation, and dual inhibition of PI3Kδ and Vps34 may lead to enhanced killing of these cells.

In summary, we have discovered a highly selective and potent PI3Kδ/Vps34 dual kinase inhibitor, PI3KD/V-IN-01, which exerts better anti-proliferative effects against the majority of B-cell related malignancies that we tested (AML, CLL and B-NHL) as compared to individual targeted PI3Kδ and Vps34 inhibitors. PI3KD/V-IN-01 is particularly potent against FLT3-ITD positive AML cells and exhibits significant anti-tumor activity *in vivo*. PI3KD/V-IN-01 thus represents a novel and potentially alternative approach to enhance the activity of PI3Kδ inhibitors and warrants further clinical investigation as a therapeutic agent for B-cell malignancies.

## MATERIALS AND METHODS

### Reagents

CAL-101, GDC-0941 and VPS34-IN-1 were purchased from Haoyuan Chemexpress Inc. (Shanghai, China) Recombinant PI3Kα, PI3Kδ, PI3Kγ, PI3KC2α, PI3KC2β, Vps34, PI4Kα, PI4Kβ and lipid substrates PI, PIP_2_:PS and PI:PS were purchased from Invitrogen. Recombinant PI3Kβ was from Sigma. The ADP-Glo™ kinase assay kit was from Promega Corporation.

### Cell lines and cell culture

The human cancer cell lines, SKM-1, Ramos and Hs 505.T were purchased from the American Type Culture Collection (ATCC) (Manassas, VA, USA). The FLT3-ITD-expressing line, MV4-11, was provided by Dr. Anthony Letai, Dana Farber Cancer Institute (DFCI), Boston, MA. The FLT3-ITD-expressing lines, MOLM-13 and MOLM-14, were provided by Dr. Scott Armstrong, DFCI, Boston, MA. The wt FLT3-expressing AML line, NB4 (KRAS A18D), was obtained from Dr. Gary Gilliland. U937, HL-60, CMK, OCI-AML-2, OCI-AML-3, HEL, HT, Namalwa, NOMO-1, MEC-1 and MEC-2 were purchased from Cobioer Biosciences CO., LTD (Nanjing, China).

### Antibodies and immunoblotting

The following antibodies were purchased from Cell Signaling Technology: Akt(#4691), Phospho-Akt(Thr308)(#4056), Phospho-Akt(Ser473)(#4060), GAPDH, GSK-3β(#9315), Phospho-GSK-3β(#9323), FoxO1(#2880), Phospho-FoxO1 (Thr24)/FoxO3a(Thr32)/FoxO4(Thr28)(#2599), PRAS40(#2691), Phospho-PRAS40 (Thr246)(#2997), 4E-BP1(#9644), p70 S6K(#2708), Phospho-p70 S6K (Thr389)(#9234). SQSTM1/p62 (D5E2)(#8025), LC3B(#12513). PARP(#9532), Caspase-3(#9665), Phospho-p44/42 MAPK (Erk1/2) (Thr202/Tyr204)(#4377), p44/42 MAPK (Erk1/2) (#4695), Phospho-NF-ΚB P65 (Ser 536)(#3033), NF-ΚB P65(#4764). Lysophosphatidic Acid (#sc-201053, Santa Cruz), Recombinant Human PDGF-BB (#220-BB, R&D), Recombinant Human C5a (Peprotech, #300-70), goat anti-IgM (#5C07615, Meridian). LC3B antibody (#3868, CST). Tubulin antibody (#8035, Santa Cruz). EEA1 antibody (#610456, BD Bioscience) and LAMP1 antibody (#24170, Abcam). Secondary antibodies, Alexa488-anti-mouse, Alexa594-anti-rabbit and Prolong gold mounting medium with DAPI were purchased from Invitrogen.

### ADP-Glo biochemical assay

The activities of PI3Kα, PI3Kβ, PI3Kδ, PI3Kγ, PI3KC2α, PI3KC2β, Vps34, PI4Kα and PI4Kβ were determined by using ADP-Glo assay, a fluorescence-based immunoassay that measures kinase activity in terms of the amount of ADP produced. A kinase titration was performed to determine the concentration. The amount of recombinant kinases was optimized to keep the reaction velocity within the linear range, to obtain an adequate difference between the resulting signals of sample in/out the presence of enzyme. The optimized enzyme concentrations for evaluating the inhibitory activities of compounds were chosen as follows: 0.16 μg/ml, 6.0 μg/ml, 1.0 μg/ml, 5.0 μg/ml, 5.0 μg/ml, 10.0 μg/ml, 1.2 μg/ml, 1.2 μg/ml and 1.0 μg/ml for PI3Kα, PI3Kβ, PI3Kδ, PI3Kγ, PI3KC2α, PI3KC2β, Vps34, PI4Kα and PI4Kβ, respectively. The ATP concentration was optimized, 10 μM ATP for PI3Kα and PI3Kβ, and 50 μM ATP for detection of the other seven kinases. The PI3Kα, PI3Kβ, PI3Kδ, PI3Kγ, PI3KC2α, PI3KC2β kinase reactions were carried out in 10 μl volume in a 384-well plate in the kinase reaction buffer containing 50 mM Hepes pH 7.5, 3 mM MgCl_2_, 1 mM EGTA, 1 mM NaCl, 0.03% CHAPS and 2 mM DTT. In the case of VPS34, 2 mM MnCl_2_ and 2 mM DTT were further added to the reaction buffer containing 50 mM Hepes pH 7.5, 4 mM MgCl_2_, 1 mM EGTA and 0.1% CHAPS. For PI4Kα and PI4Kβ, the reaction buffer consisting of 50 mM Tris pH 7.5, 5 mM MgCl_2_, 0.5 mM EGTA, 0.4% Triton X-100 and 2 mM DTT was used. In all cases, 2.5 μl of the respective kinase was added and the mixture was incubated at room temperature for 1 hour in the presence or absence of various concentrations of a given inhibitor. Each reaction was initiated by the addition of 2.5 μl mixture of optimized concentrations of ATP and the substrate (50 μM PIP2:PS for PI3Kα, PI3Kβ, PI3Kδ and PI3Kγ; 100 μM PI:PS for VPS34, PI4Kα and PI4Kβ; 100 μM PI for PI3KC2α and PI3KC2β, respectively). The assay was proceeded for 1 hour at 37 °C before addition of 5 μl ADP-Glo reagent, and incubated for 40 min at room temperature. 10 μl kinase detection reagent was dded and incubated for 30 min at room temperature, before the luminescence signal was read with an envision PerkinElmer plate reader.

### Kinase kinetic assay

Kinetic analyses of PI3Kδ and Vps34 were performed using a luminometric kinase assay varying the concentration of ATP using the ADP-Glo reagents (Promega). The serially diluted PI3KD/V-IN-01 and PI3Kδ (1.0 μg/mL) were assayed in a reaction (10 μL) containing 50 mM Hepes pH 7.5, 3 mM MgCl_2_, 1 mM EGTA, 1 mM NaCl, 0.03% CHAPS, 2 mM DTT, and Vps34 (1.2 μg/mL). In the case of Vps34, 2 mM MnCl2 and 2 mM DTT were further added to the reaction buffer containing 50 mM Hepes pH 7.5, 4 mM MgCl2, 1 mM EGTA and 0.1% CHAPS. After 60 min incubation at RT, varied concentrations of ATP and 0.1 mM substrate (PIP2:PS for PI3Kδ, PI:PS for Vps34) were added and incubated for 60 min at 37° C. The overall rate of reaction was determined as the slope of the decreasing phase of the reaction. Each data point was collected in duplicate and kinetic parameters were obtained using Prism 5.0 (GraphPad Software, San Diego, CA).

### PI3K isoforms cellular selectivity assay

NIH-3T3(ATCC), NIH-3T3, RAW264.7 (ATCC) macrophages and Raji (ATCC) cells were seeded in a 6-well tissue culture plate and starved for 24 hours, then incubated with PI3KD/V-IN-01 at the desired concentrations for 1 hour followed by 20 ng/ml PDGF-BB for 10 min, 5μM LPA for 10 min, 50 ng/ml c5a for 5 min, and 1μg/ml anti-IgM for 10 min. Cells were lysed and AKT phosphorylation was determined by Western Blotting. Intensity of the bands was determined using ImageJ 1.42q (NIH, USA) and normalized to total AKT (loading control).

### Drug combination studies

The cell lines, OCI-AML-2, MEC-2, Namalwa, and MV4-11, were grown in 96-well culture plates (3000/well). A combination of the PI3K kinase inhibitor CAL101 with the VPS34 inhibitor VPS34-IN-1, was used to treat the cell lines. Cell proliferation were determined after treatment with the compounds for 72 hours. Cell viability was measured using the CellTiter–Glo assay (Promega, USA), data were normalized to control groups (DMSO) and represented by the mean of three independent measurements with a standard error <20%.

### Autophagy studies

HeLa cells were treated with different concentrations of inhibitors for 16 hours. For all immunofluorescence experiments, cells were fixed in -20 °C methanol for 5 minutes or 3.7% (v/v) formaldehyde in phosphate-buffered saline (PBS) for 20 minutes. For LC3B staining, cells were fixed in -20 °C methanol. For EEA1 and LAMP1 staining, cells were fixed in PBS+ formaldehyde. Cells were then permeabilized with TBSTx (0.1% Triton X-100), blocked in AbDil (TBSTx + 2% BSA + 0.1% NaN3), and probed with primary and secondary antibodies diluted in AbDil. Cells were rinsed thoroughly with TBSTx and mounted by anti-fade prolong Gold with DAPI. Images were taken using a Leica DMI4000B fluorescent microscope. All experiments were repeated at least three times). MEC-2, OCI-AML-2, OCI-AML-3 and MV4-11 cells were treated with serially-diluted PI3KD/V-IN-01 for 6/12 hours. Hela cells were starved with EBSS for 2 hours, then treated with 25μM HCQ and serially diluted PI3KD/V-IN-01 for 1 hour. Cells were lysed in lysis buffer. P62, LC3B, and GAPDH antibodies were used for immunoblotting.

### Primary cells and primagraft cells

Mononuclear cells were isolated from AML patients. Mononuclear cells were isolated by density gradient centrifugation through Ficoll-Plaque Plus (Amersham Pharmacia Biotech AB, Uppsala, Sweden) at 2000 rpm for 30 minutes, followed by two washes in 1X PBS. Freeze-thawed cells were then cultured in liquid culture (DMEM, supplemented with 20% FBS). All blood and bone marrow samples from AML patients were obtained through written consent under approval of the Dana Farber Cancer Institute Institutional Review Board. The ethics committees approved the consent procedure.

Peripheral blood mononuclear cells (PBMCs) from individuals with CLL were isolated by density centrifugation through Ficoll and frozen for each subject. Those subjects with low white counts whose CLL cell purity was expected to be < 85% underwent B cell isolation using RosetteSep. The protocol was approved by the Dana-Farber Harvard Cancer Center Institutional Review Board and all subjects signed written informed consent prior to participation.

### MV4-11 xenograft tumor model

Six week old female nu/nu mice were purchased from the Shanghai Experimental Center, Chinese Science Academy (Shanghai). All animals were maintained in a specific pathogen-free facility and used according to the animal care regulations of Hefei Institutes of Physical Science Chinese Academy of Sciences, and all efforts were made to minimize animal suffering. To obtain an orthotopic xenograft of human mammary tumor in the mice, cells were harvested during exponential growth. 7 million MV4-11 cells in PBS were suspended in a 1:1 mixture with Matrigel (BD Biosciences) and injected into the subcutaneous space on the right flank of nu/nu mice. Daily intraperitoneal injection was initiated when MV4-11 tumors had reached a size of 200 to 400 mm^3^. Animals were then randomized into treatment groups of 5 mice each for efficacy studies. Compound PI3KD/V-IN-01 was delivered daily in a HKI solution (0.5% Methocellulose/0.4% Tween80 in ddH_2_O) by orally gavages. A range of doses of compound PI3KD/V-IN-01 or its vehicle was administered, as indicated in the figure legends. Body weight and tumor growth were measured daily after PI3KD/V-IN-01 treatment. Tumor volumes were calculated as follows: tumor volume (mm^3^)=[(W^2^× L)/2] in which width (W) is defined as the smaller of the two measurements and length(L) is defined as the larger of the two measurements.

### TUNEL staining

TUNEL staining was performed using the POD in Situ Cell Death Detection kit (Roche, USA). Sections were deparaffinized in xylene, rehydrated in decreasing concentrations of ethanol, then treated by nuclease free Proteinase K for 15 min at room temperature before endogenous peroxidase was blocked in 3% H_2_O_2_ in methanol. Terminal deoxynucleotidyl transferase (TdT) in reaction buffer was applied to sections for 1h at 37°C. Following washes, the slides were covered by converter-POD solution for 30 min at 37°C. Apoptotic cells were detected after incubation in the 3, 3′-diaminobenzidine (DAB) chromogen (Beyotime Biotechnology) for approximately 8 min and slides were counterstained with hematoxylin.

### Ki-67 staining

For IHC demonstration of Ki-67, tissue sections were quenched for endogenous peroxides and placed in an antigen retrieval solution (0.01M citrate buffer, PH 6.0) for 15 min in a microwave oven at 100°C at 600W. After incubation in the casein block, mouse MAb anti-Ki-67 (ZSGB-BIO) was applied to the sections at dilutions of 1:50. Incubations with primary antibodies lasted overnight at 4°C. The secondary detection system was used to visualize antibody binding. Staining was developed with DAB; slides were counterstained with hematoxylin, dehydrated and mounted.

### HE staining

HE staining was carried out as previously described.[15] First, the sections were hydrated and then the slide was dipped into a Coplin jar containing Mayer's hematoxylin and agitated for 30 sec. The slide was then rinsed in H_2_O for 1 min. The slide was then stained with 1% eosin Y solution for 10-30sec with agitation. Subsequently, the sections were rinsed with two changes of 95% alcohol and two changes of 100% alcohol for 30 sec each. The alcohol was then extracted with two changes of xylene. Finally, one or two drops of mounting medium was added prior to covering with a cover slip.

## SUPPLEMENTARY FIGURES AND TABLE



## References

[1] Chantry D, Vojtek A, Kashishian A, Holtzman DA, Wood C, Gray PW, Cooper JA, Hoekstra MF (1997). p110delta, a novel phosphatidylinositol 3-kinase catalytic subunit that associates with p85 and is expressed predominantly in leukocytes. J Biol Chem.

[2] Fruman DA, Rommel C (2011). PI3Kdelta inhibitors in cancer: rationale and serendipity merge in the clinic. Cancer Discov.

[3] Billottet C, Grandage VL, Gale RE, Quattropani A, Rommel C, Vanhaesebroeck B, Khwaja A (2006). A selective inhibitor of the p110delta isoform of PI 3-kinase inhibits AML cell proliferation and survival and increases the cytotoxic effects of VP16. Oncogene.

[4] Bernal A, Pastore RD, Asgary Z, Keller SA, Cesarman E, Liou HC, Schattner EJ (2001). Survival of leukemic B cells promoted by engagement of the antigen receptor. Blood.

[5] Brown JR, Byrd JC, Coutre SE, Benson DM, Flinn IW, Wagner-Johnston ND, Spurgeon SE, Kahl BS, Bello C, Webb HK, Johnson DM, Peterman S, Li D, Jahn TM, Lannutti BJ, Ulrich RG (2014). Idelalisib, an inhibitor of phosphatidylinositol 3-kinase p110delta, for relapsed/refractory chronic lymphocytic leukemia. Blood.

[6] Vanhaesebroeck B, Khwaja A (2014). PI3Kdelta inhibition hits a sensitive spot in B cell malignancies. Cancer cell.

[7] Fruman DA, Rommel C (2014). PI3K and cancer: lessons, challenges and opportunities. Nat Rev Drug Discov.

[8] Shanware NP, Bray K, Abraham RT (2013). The PI3K, metabolic, and autophagy networks: interactive partners in cellular health and disease. Annu Rev Pharmacol Toxicol.

[9] Mahoney E, Lucas DM, Gupta SV, Wagner AJ, Herman SE, Smith LL, Yeh YY, Andritsos L, Jones JA, Flynn JM, Blum KA, Zhang X, Lehman A, Kong H, Gurcan M, Grever MR (2012). ER stress and autophagy: new discoveries in the mechanism of action and drug resistance of the cyclin-dependent kinase inhibitor flavopiridol. Blood.

[10] Axe EL, Walker SA, Manifava M, Chandra P, Roderick HL, Habermann A, Griffiths G, Ktistakis NT (2008). Autophagosome formation from membrane compartments enriched in phosphatidylinositol 3-phosphate and dynamically connected to the endoplasmic reticulum. J Cell Biol.

[11] Bago R, Malik N, Munson MJ, Prescott AR, Davies P, Sommer E, Shpiro N, Ward R, Cross D, Ganley IG, Alessi DR (2014). Characterization of VPS34-IN1, a selective inhibitor of Vps34, reveals that the phosphatidylinositol 3-phosphate-binding SGK3 protein kinase is a downstream target of class III phosphoinositide 3-kinase. Biochem J.

[12] Ronan B, Flamand O, Vescovi L, Dureuil C, Durand L, Fassy F, Bachelot MF, Lamberton A, Mathieu M, Bertrand T, Marquette JP, El-Ahmad Y, Filoche-Romme B, Schio L, Garcia-Echeverria C, Goulaouic H (2014). A highly potent and selective Vps34 inhibitor alters vesicle trafficking and autophagy. Nat Chem Biol.

[13] Dowdle WE, Nyfeler B, Nagel J, Elling RA, Liu S, Triantafellow E, Menon S, Wang Z, Honda A, Pardee G, Cantwell J, Luu C, Cornella-Taracido I, Harrington E, Fekkes P, Lei H (2014). Selective VPS34 inhibitor blocks autophagy and uncovers a role for NCOA4 in ferritin degradation and iron homeostasis *in vivo*. Nat Cell Biol.

[14] Yu X, Long YC, Shen HM (2015). Differential Regulatory Functions of Three Classes of Phosphatidylinositol and Phosphoinositide 3-Kinases in Autophagy. Autophagy.

[15] Fischer AH, Jacobson KA, Rose J, Zeller R (2008). Hematoxylin and eosin staining of tissue and cell sections. CSH Protoc.

[16] Carpentier S, N'Kuli F, Grieco G, Van Der Smissen P, Janssens V, Emonard H, Bilanges B, Vanhaesebroeck B, Gaide Chevronnay HP, Pierreux CE, Tyteca D, Courtoy PJ (2013). Class III phosphoinositide 3-kinase/VPS34 and dynamin are critical for apical endocytic recycling. Traffic.

[17] van Dam EM, Ten Broeke T, Jansen K, Spijkers P, Stoorvogel W (2002). Endocytosed transferrin receptors recycle via distinct dynamin and phosphatidylinositol 3-kinase-dependent pathways. The Journal of biological chemistry.

