# Supplementary material for: Simultaneous inhibition of Vps34 kinase would enhance PI3Kδ inhibitor cytotoxicity in the B-cell malignancies
Source: Oncotarget. 2016 Jul 18;7(33):53515–25. doi: 10.18632/oncotarget.10650 (PMC5288202; doi:10.18632/oncotarget.10650)
Supplement: Supplementary file 1 [file oncotarget-07-53515-s001.pdf]

# Simultaneous inhibition of Vps34 kinase would enhance PI3Kδ inhibitor cytotoxicity in the B-cell malignancies

## SUPPLEMENTARY DATA

### Chemical synthesis of PI3KD/V-IN-1

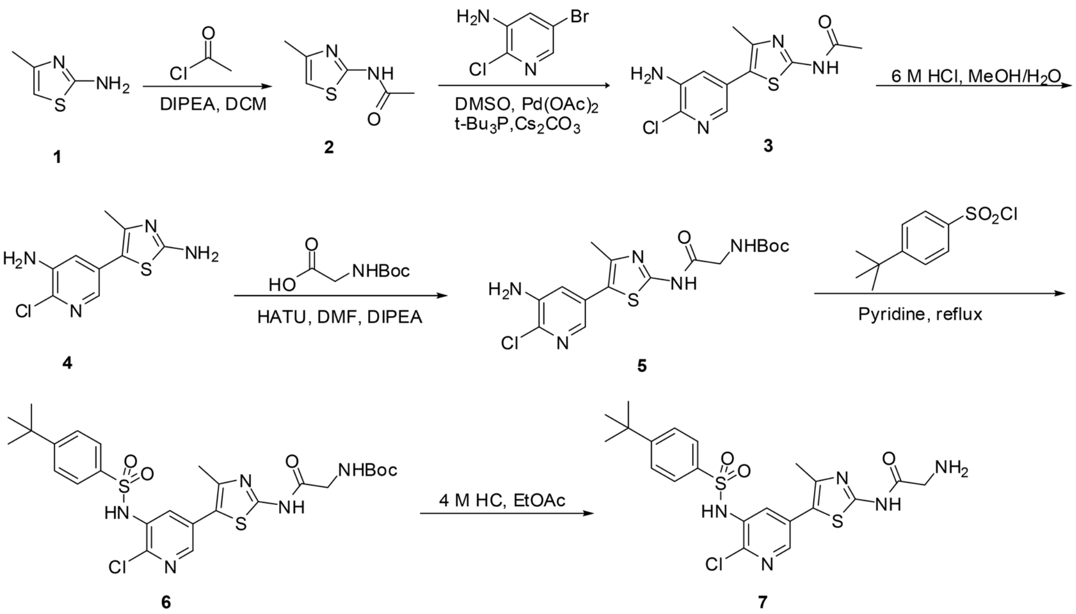

### Chemical synthesis

TOF LC-MS analysis was conducted on an Agilent 1260 HPLC with a quadrupole mass analyzer. LC chromatography used an Agilent XDB-C18 column (4.6 × 50 mm, 1.8 µm) with a water/acetonitrile (each with 0.2% (v/v) formic acid) gradient at a flow rate of 0.4 mL/min. $^{1}$H and $^{13}$C spectra were recorded at 400 and 100 MHz, respectively, on a Bruker Spectrometer. Column chromatography was conducted using either silica gel (Silicycle 40−64 µm). All moisture-sensitive reactions were carried out using dry solvents under a slight pressure of ultra pure quality argon. Glassware was dried in an oven at 140 °C for at least 12 h prior to use, and it was then assembled quickly while hot, sealed with rubber septa, and allowed to cool under a stream of argon. Reactions were stirred magnetically using Teflon-coated magnetic stirring bars. Commercially available disposable syringes were used for transferring the reagents and solvents. All reagents were used as received from commercial sources, unless specified otherwise, or prepared as described in the literature. The purities of all compounds were determined to be >95% by HPLC.

### 4-methylthiazol-2-amine(2)

To a solution of 2-amino-4-methylthiazole(15 g, 131.4 mmol, 1.00 equiv) in DCM (100 mL) was added TEA (24 mL, 144.5 mmol, 1.10 equiv) at 0 °C under argon. Then a solution of acetyl chloride (9.8 mL, 138.0 mmol, 1.05 equiv) in DCM (20 mL) was slowly added. The reaction mixture was stirred at 0 °C for 1h, then it was allowed to warm to room temperature for 14h. The resulting mixture was diluted with DCM (100 mL), washed with water (2×50 mL), brine (50 mL) and dried over (anhydrous $MgSO_4$). Evaporation of the solvent was afforded the crude product, which was crystallized from DCM to give 4-methylthiazol-2-amine as off white solid.$^{1}$H NMR (400 MHz, DMSO-d6) δ 12.04 (s, 1H), 6.71 (s, 1H), 2.25 (s, 3H), 2.13 (s, 3H).$^{13}$C NMR (101 MHz, DMSO-d6) δ 168.63, 157.79, 146.96, 107.85, 22.93, 17.33. TOF LCMS(m/z):157.0342[M+H]$^{+}$.

### N-(5-(5-amino-6-chloropyridin-3-yl)-4-methylthiazol-2-yl)acetamide(3)

To a solution of 3-amino-5-bromo-2-chloropyridine (8.3 g, 40.0 mmol, 1.00 equiv) in DMSO (80 mL)

was added **2** (10.0 g, 64.0 mmol, 1.6 equiv) at room temperature under argon. Then CsF(21.5 g), $Pd(OAc)_2$ (1.03 g) and t-$Bu_3P$ (20 mL, 0.2 M in heptane) was added. The reaction mixture was degassed by argon. Then it was heated to 130 °C for 48h. The resulting mixture was cooled to room temperature and diluted with ice-cold water (1L). The precipitate was filtered and washed with water. The precipitate was dissolved in DCM/MeOH (1:1) (100 mL) and decolored by activated carbon. The yellow solid was diluted with diethyl ether (100 mL), and the yellow was filtered and dried to provide N-(5-(5-amino-6-chloropyridin-3-yl)-4-methylthiazol-2-yl)acetamide.$^{1}$H NMR (400 MHz, DMSO-d6) δ 7.68 (s, 1H), 7.25 (s, 1H), 5.75 (s, 2H), 2.35 (s, 3H), 2.15 (s, 3H).$^{13}$C NMR (101 MHz, DMSO-d6) δ 169.28, 156.64, 143.65, 141.78, 135.28, 133.97, 129.06, 121.31, 120.01, 23.07, 16.64. TOF LCMS(m/z):283.0471[M+H]$^+$.

**5-(5-amino-6-chloropyridin-3-yl)-4-methylthiazol-2-amine(4)**

To a solution of N-(5-(5-amino-6-chloropyridin-3-yl)-4-methylthiazol-2-yl)acetamide ( 2. 00 g, 0.71 mmol, 1.00 equiv) in EtOH (40 mL) was added 6 M HCl solution ( 7 mL) at room temperature. Then the reaction mixture was heated to reflux for 5h. The resulting mixture was concentrated to dryness. The residue was diluted with EtOH and filtered to afford the 5-(5-amino-6-chloropyridin-3-yl)-4-methylthiazol-2-amine. $^{1}$H NMR (400 MHz, DMSO-d6) δ 9.77 (s, 2H), 7.65 (s, 1H), 7.29 (s, 1H), 2.27 (s, 3H).$^{13}$C NMR (101 MHz, DMSO-d6) δ 168.00, 141.64, 135.22, 132.82, 126.33, 121.44, 112.78, 13.23. TOF LCMS(m/z):241.0239[M+H]$^+$.

**tert-butyl2-(5-(5-amino-6-chloropyridin-3-yl)-4-methylthiazol-2-ylamino)-2-oxoethylcarbamate(5)**

To a solution of 5-(5-amino-6-chloropyridin-3-yl)-4-methylthiazol-2-amine (80 mg, 0.21 mmol, 1.00 equiv) in anhydrous DMF (2 mL) was added Boc-Glcycine (37 mg, 0.21 mmol, 1.00 equiv), HATU (80 mg, 0.21 mmol, 1.00 equiv) and DIPEA (0. 17 mL, 1.00 mmol, 5.0 equiv) at 0 °C under argon. The reaction mixture was stirred at 0 °C for 1 hour then was allowed to warm to room temperature for 20 hours. The resulting mixture was concentrated to dryness. The residue was diluted with water (30 mL), extracted with EtOAc(3×30 mL). The combined organic layers were washed with water(50 mL), brine (50 mL) and dried over( anhydrous $Na_2SO_4$). Evaporation of the solvent was provided the crude product, which was purified by flash chromatography (eluting with MeOH in DCM 0-1.5%) to give the compound tert-butyl 2-(5-(5-amino-6-chloropyridin-3-yl)-4-methylthiazol-2-ylamino)-2-oxoethylcarbamate as yellow solid ( 54 mg, yield 65%).$^{1}$H NMR (400 MHz, DMSO-d6) δ 12.26 (s, 1H), 7.69 (s, 1H), 7.22 (s, 1H), 3.86 (s, 2H), 2.36 (s, 3H), 1.39 (s, 9H).$^{13}$C NMR (101 MHz, DMSO-d6) δ 169.16, 156.34, 155.95, 143.90, 141.79, 135.32, 134.16, 128.82, 121.31, 120.42, 78.87, 54.05, 28.34, 17.07. TOF LCMS(m/z):398.0970[M+H]$^+$.

**tert-butyl2-(5-(5-(4-tert-butylphenylsulfonamido)-6-chloropyridin-3-yl)-4-methylthiazol-2-ylamino)-2-oxoethylcarbamate(6)**

To a solution of tert-butyl 2-(5-(5-amino-6-chloropyridin-3-yl)-4-methylthiazol-2-ylamino)-2-oxoethylcarbamate (70 mg, 0.18 mmol, 1.00 equiv) in anhydrous pyridine (3 mL) was added 4-tert-butylbenzensulfonyl chloride (81 mg, 0.35 mmol, 2.00 equiv) at room temperature under argon. Then the reaction mixture was heated to reflux for 20 hours. The resulting mixture was concentrated to give the crude product, which was purified by flash chromatography (eluting with MeOH in DCM 0-2%) to give tert-butyl 2-(5-(5-(4-tert-butylphenylsulfonamido)-6-chloropyridin-3-yl)-4-methylthiazol-2-ylamino)-2-oxoethylcarbamate as white solid 53 mg, yield 50%.$^{1}$H NMR (400 MHz, $CDCl_3$) δ 8.16 (s, 1H), 7.99 (s, 1H), 7.77 (d, $J$ = 8.2 Hz, 2H), 7.52 (d, $J$ = 8.4 Hz, 2H), 4.32 (t, $J$ = 6.6 Hz, 2H), 2.34 (s, 3H), 1.47 (s, 9H), 1.31 (s, 9H).$^{13}$C NMR (101 MHz, $CDCl_3$) δ 168.26, 157.93, 156.55, 156.23, 144.43, 144.15, 140.33, 135.57, 132.32, 130.90, 128.84, 127.10, 126.51, 120.29, 65.58, 35.03, 31.84, 30.88, 28.30, 13.36. TOF LCMS(m/z):594.1531[M+H]$^+$.

**2-amino-N-(5-(5-(4-tert-butylphenylsulfonamido)-6-chloropyridin-3-yl)-4-methylthiazol-2-yl)acetamide(7)**

To a solution of tert-butyl2-(5-(5-(4-tert-butylphenylsulfonamido)-6-chloropyridin-3-yl)-4-methylthiazol-2-ylamino)-2-oxoethylcarbamate (20 mg, 0.034 mmol, 1.00 equiv) in EtOAc (1 mL) was added 1 mL of 4 M HCl ( in EtOAc) at room temperature. The reaction mixture was stirred at room temperature for 1h. The resulting precipitate was filtered and washed with EtOAc to afford the 2-amino-N-(5-(5-(4-tert-butylphenylsulfonamido)-6-chloropyridin-3-yl)-4-methylthiazol-2-yl)acetamide as white solid. $^{1}$H NMR (400 MHz, $CD_3OD$) δ 8.2 (s, 1H), 8.00 (s, 1H), 7.82 – 7.75 (m, 2H), 7.66 – 7.60 (m, 2H), 4.14 (s, 2H), 2.40 (s, 3H), 1.35 (s, 9H).$^{13}$C NMR (101 MHz, $CD_3OD$) δ 163.60, 155.74, 142.94, 141.52, 135.29, 131.26, 130.04, 129.45, 126.97, 126.43, 125.25, 124.57, 118.44, 39.39, 33.16, 28.44, 12.71. TOF LCMS(m/z):494.1198[M+H]$^+$.

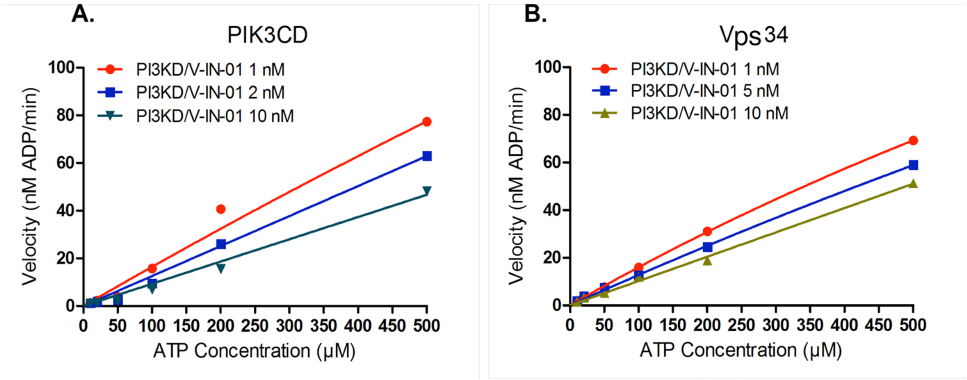


**Supplementary Figure S1: Kinetic study of PI3KD/V-IN-01 against PI3Kδ and Vps34.**

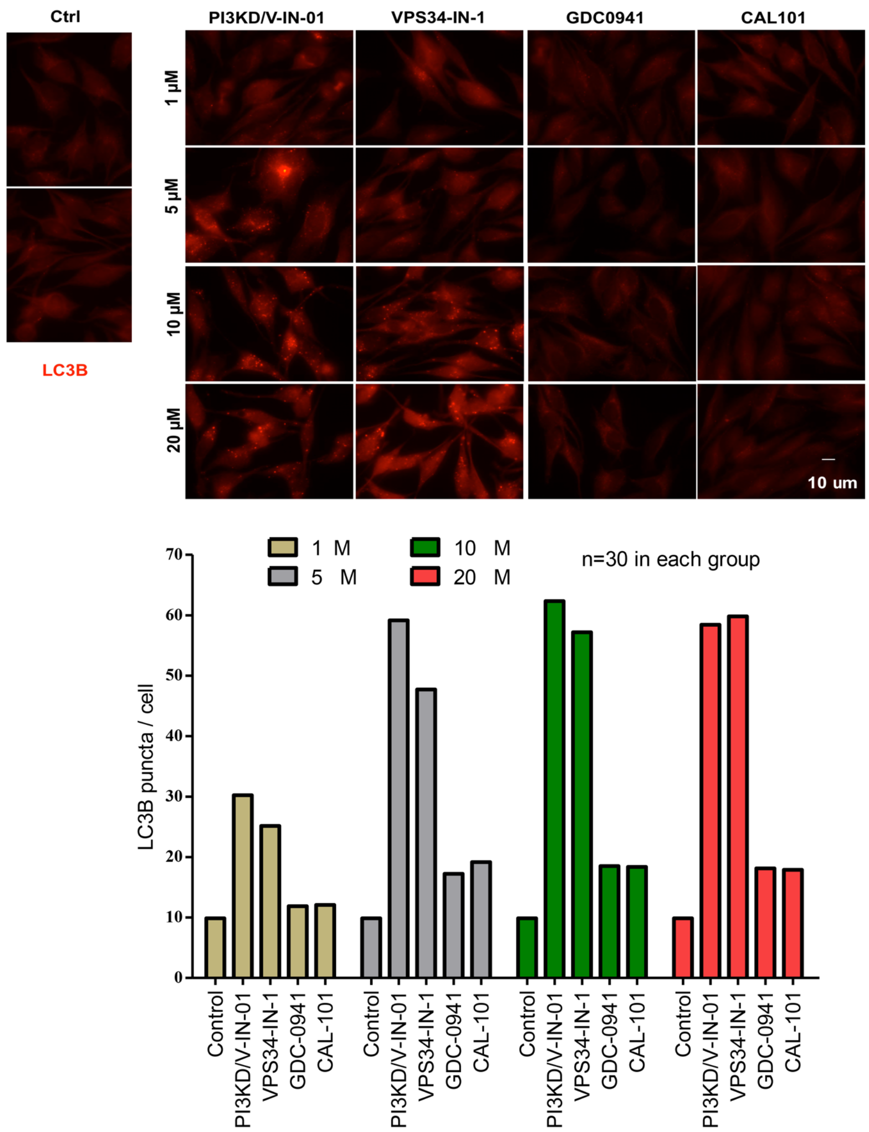


**Supplementary Figure S2: PI3KD/V-IN-01 affects autophagy HeLa cells were treated with different concentrations of PI3KD/V-IN-01, VPS34-IN-1, GDC-0941 or CAL-101 for 16 hours before they were fixed and stained for the autophagy marker LC3B.** Scale bar: 10 µM.

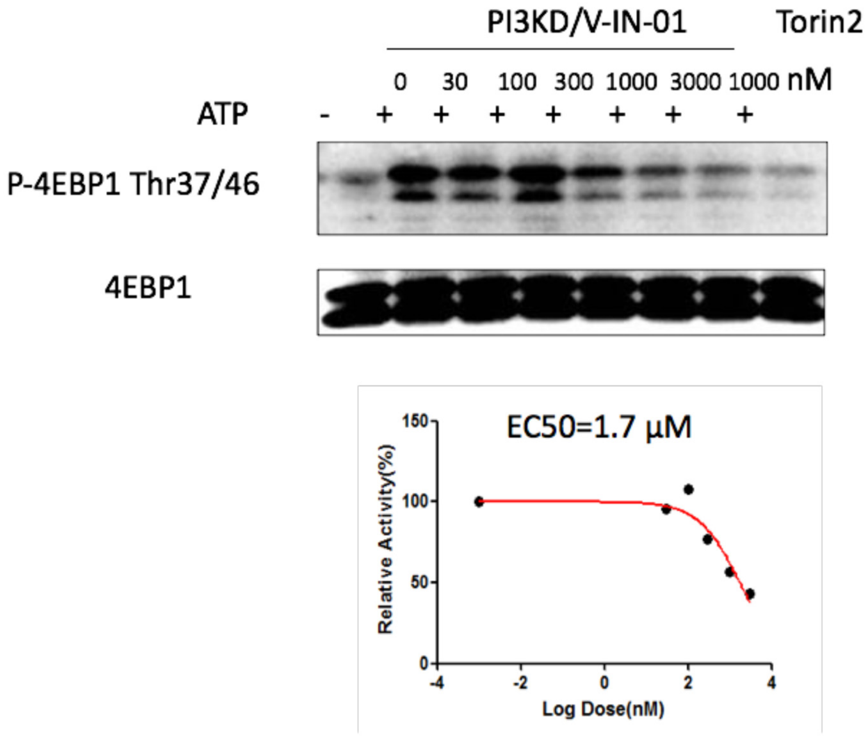


**Supplementary Figure S3: In vitro IP kinase assay of PI3KD/V-IN-01 against mTORC1.**

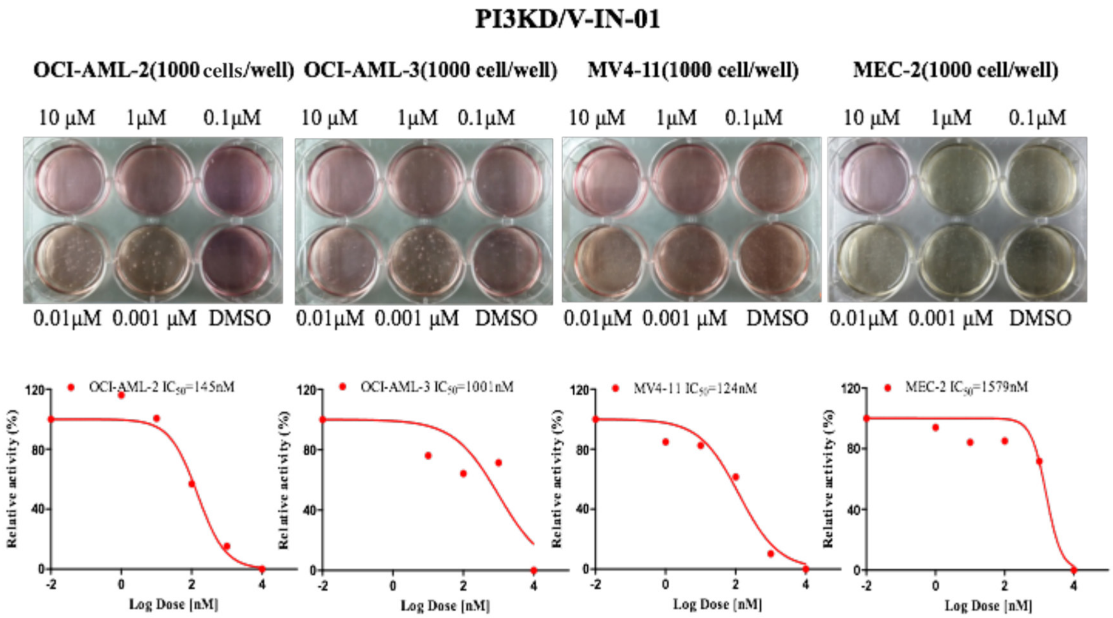


**Supplementary Figure S4: Colonogenic assay of PI3KD/V-IN-01 against four B-cell cancer cell lines.**

**Supplementary Table S1: Patient primary cell information**

| | Age | Gender | % blasts | Pathology | Cytogenetics |
|---|---|---|---|---|---|
| AML6 | 79 | F | 88 | AML | 45,XX,-3,del(5)(q13q33),-7, add(9)(q22),del(12)(q11.2q21), add(13)(q22),del(20)(q11.2),+mar[cp10] |
| AML7 | 42 | F | 22 | AML | 46-47,XX,add(2)(p11.2),+16, r(16)idic(16)(q11.2)(p13;p13)x1-2,der(17)[cp4]/46,XX[5] |
| AML8 | pediatric | M | not available | AML | 46,XY,add(6)(q21),add(9)(p24)[10]/46,XY[10] |
| AML9 | 75 | M | 6 | AML | 47, XY, +11 |
